# Non-Contrast Magnetic Resonance Lymphography: Technical Approach Toward Protocol Standardization

**DOI:** 10.3390/jcm15218589

**Published:** 2026-11-04

**Authors:** Theodoros Chrelias, Evangelos Dimakakos, Konstantinos Samiotis, Kalliopi Varaki, Margarita Toumanidou, Kyriaki Cholidou, Urska Bregar, Peter Marschang, Chris Manu, Nikolaos Michalopoulos, Nikolaos Arkadopoulos, Nikolaos Papadopoulos

**Affiliations:** 1 Department of Plastic, Reconstructive and Aesthetic Surgery, Hospital Sud, University of Rennes, 35203 Rennes, France; 2 School of Medicine, National and Kapodistrian University of Athens, 11527 Athens, Greecenikolpapadop@med.uoa.gr (N.P.); 3 Center of Prevention, Diagnosis and Treatment of Lymphedema—Lymphatic Diseases for Adults and Children, Metropolitan Hospital, 18547 Athens, Greece; edimakakos@uniwa.gr; 4 Department of Anatomy and Physiology, School of Nursing, University of West Attica, 12210 Athens, Greece; 5 VAS-European Independent Foundation in Angiology / Vascular Medicine, 20157 Milan, Italy; urska.bregar@kclj.si (U.B.); peter.marschang@sabes.it (P.M.);; 6 Department of Medical Imaging, Metropolitan Hospital, 18547 Athens, Greece; 7 KSAM Medical Imaging Center Ltd., 2057 Nicosia, Cyprus; 8 Department of Radiology, Metropolitan Hospital, 18547 Piraeus, Greece; 9 First University Department of Respiratory Medicine, Thoracic Diseases General Hospital “Sotiria”, 11527 Athens, Greece; 10 Department of Vascular Diseases, Division of Internal Medicine, University Medical Centre Ljubljana, Zaloška cesta 7, 1000 Ljubljana, Slovenia; 11 Department of Internal Medicine, Central Hospital of Bolzano (SABES-ASDAA), 39100 Bolzano, Italy; 12 Department of Diabetic Foot Medicine, King’s College Hospital NHS Foundation Trust, Denmark Hill, London SE5 9RS, UK; 13 1st Propaedeutic Department of Surgery, Hippokration General Hospital of Athens, 11527 Athens, Greece; 14 4th Department of Surgery, Attikon University General Hospital, 12462 Athens, Greece

**Keywords:** lymphedema, non-contrast magnetic resonance lymphangiography, lymphatic system imaging, lymphatic vessels, lower extremities

## Abstract

**Background:** Lymphedema is a chronic condition for which accurate imaging evaluation of the lymphatic system can contribute to diagnosis, disease assessment, and treatment planning. Non-contrast magnetic resonance lymphangiography (NCMRL) is an imaging technique that enables morphological visualization of lymphatic abnormalities without contrast agent administration. This technical report aims to describe the NCMRL protocol routinely implemented at our institution, with particular emphasis on lower-extremity imaging. **Methods:** The institutional NCMRL workflow performed on a 3 Tesla (3T) magnetic resonance imaging (MRI) system is described, including patient positioning, three-station anatomical coverage, pulse sequence selection, acquisition parameters, geometric alignment, and image post-processing using segmental and panoramic maximum intensity projection (MIP) reconstructions. **Results:** The report documents the acquisition and post-processing workflow and illustrates its imaging output through representative clinical images. No systematic assessment of diagnostic accuracy, quantitative image quality, or examination success rates was performed. **Conclusions:** This technical report provides a structured description of an existing institutional NCMRL workflow for lower-extremity imaging. The documented approach may serve as a reference for future protocol optimization and standardization, while its reproducibility and broader clinical applicability require further evaluation.

## 1. Introduction

Lymphedema is defined as pathological swelling caused by the accumulation of lymphatic fluid in various parts of the body due to impaired lymphatic drainage [1]. The evaluation of lymphedema through diagnostic examinations helps not only to confirm the diagnosis and determine its severity but also to design an appropriate treatment plan.

This evaluation may be achieved in a variety of ways. Lymphoscintigraphy is currently considered the gold standard for imaging the lymphatic system [2]. However, the technique used in lymphoscintigraphy has limitations due to its low spatial and temporal resolution, and it is typically limited to producing two-dimensional projection images of the major lymphatic pathways [3,4,5].

Due to the limitations of lymphoscintigraphy, there has been growing scientific interest in magnetic resonance lymphangiography in recent years, as it has emerged as a non-invasive alternative, offering high soft-tissue resolution and detailed imaging of the lymphatic network.

However, despite advances in MRI techniques for the lymphatic system, there remains significant heterogeneity in imaging protocols, particularly with regard to non-contrast-enhanced techniques. Differences in sequences, parameters, and image reconstruction methods limit reproducibility across different centers.

Non-contrast magnetic resonance lymphangiography (NCMRL) is primarily based on heavily T2-weighted sequences and allows for the visualization of lymphatic pools and arranged lymphatic vessels without the use of contrast agents [6].

The need for standardization of protocols remains critical, as the lack of standardization limits widespread clinical application and the ability to compare results across different studies and centers.

The purpose of this study is to provide a detailed description of the imaging technique for the lymphatic system, with particular emphasis on the lymphatic vessels and lymphatic pathways of the lower extremities, using non-contrast magnetic lymphangiography. At the same time, the description of this specific methodology aims to contribute to the ongoing effort to optimize and standardize the examination, with the ultimate goal of establishing common protocols for its application in the future.

## 2. Materials and Methods

This study was exclusively technical in scope and focused on the detailed description of the MRI acquisition parameters and imaging workflow routinely implemented for NCMRL at our institution. The investigation concerned the imaging technique and protocol itself rather than a medical or experimental intervention involving the examined individuals. No additional invasive procedure, administration of contrast agents or investigational medication, or other research-related intervention was performed.

The present work was not designed as a comparative study or a diagnostic accuracy investigation. Its primary objective was to document the NCMRL protocol and to propose this technical workflow as a potential framework for future standardization.

The images presented in this technical report were obtained during routine clinical examinations performed at our institution and were selected as representative examples to illustrate the described NCMRL acquisition and post-processing workflow. The manuscript does not report a prospectively recruited patient cohort or a systematic clinical outcome analysis. All images included in this report were anonymized prior to publication. The images were selected to illustrate the anatomical coverage, complementary imaging sequences, and post-processing steps of the described workflow, without a systematic review of a predefined number of examinations.

### 2.1. Equipment and Hardware Configuration

All non-contrast magnetic resonance lymphangiography (NCMRL) examinations were performed on a 3 Tesla clinical MRI scanner (Magnetom Skyra, Siemens Healthcare GmbH, Erlangen, Germany.) operating on the MR XA60 platform (Full Software Version: VB60S_HF02, Release Version: VB60). High parallel imaging acceleration and deep signal penetration were achieved using an integrated multi-channel coil architecture. To facilitate comprehensive, multi-station coverage without requiring manual coil repositioning, a combined array of dedicated coils was activated simultaneously. This specific hardware configuration utilized the precise elements of the Body matrix coil (Body 30 3T) and the Spine matrix coil.

### 2.2. Patient Positioning and Somatotype Adaptations

Subject positioning was strictly dynamic, tailored to the patient’s clinical compliance, physical limitations, and structural somatotype to maintain optimal target centering near the magnet’s isocenter, thereby maximizing main magnetic field (B0) homogeneity and minimizing geometric distortion.

Lower Extremities: Patients were uniformly scanned in the Feet First-Supine (FFS) position. Both limbs were elevated on a dedicated foam ramp stabilizer, aligning them parallel to the main magnetic field (B0). Longitudinal coverage was mapped via a 3-station technique based on patient stature, capturing the pedal/distal crural zone (Station 1), the proximal crural and knee zone (Station 2), and the femoral-inguinal/pelvic girdle (Station 3).

Technical Limitations & Patient Variance: In overweight or large-framed patients, localized (B0) and (B1) field inhomogeneities frequently induced partial or incomplete fat suppression, particularly within the pelvic girdle. Furthermore, direct physical contact between the patient’s body and the magnet bore wall occasionally introduced susceptibility artifacts and localized signal degradation, necessitating careful monitoring during image interpretation.

### 2.3. Parameter Standardization and Spatial Co-Registration

To facilitate multiplanar anatomical correlation and cross-referencing of imaging findings, consistent geometric settings were applied to corresponding acquisitions within the institutional imaging workflow. Acquisition geometry was defined separately for each sequence, with coronal orientation for 3D T2 SPACE STIR and T1 TIRM and axial orientation for T2 TIRM. Table 1 reports slice thicknesses of 3.0 mm for 3D T2 SPACE STIR and 4.0 mm for the TIRM sequences. As indicated in the table footnote, the field of view was adjusted according to patient anatomy and the required anatomical coverage.

The use of compatible geometric settings facilitated spatial correspondence and anatomical comparison of imaging findings between axial and coronal datasets.

### 2.4. Pulse Sequence Suite and Post-Processing Pipeline

The non-contrast imaging suite utilized a synergistic combination of heavy T2-weighted anatomical sequences optimized for high-resolution fluid mapping:

Anatomy and Fluid Mapping: Coronal 3D T2 Sampling Perfection with Application-optimized Contrasts using different flip angle Evolutions (SPACE) with Short Tau Inversion Recovery (STIR) (repetition time [TR]: 4000 ms, echo time [TE]: 350 ms, inversion time [TI]: 200 ms) was deployed as the cornerstone sequence for high-resolution fluid-sensitive reconstructions. This was supported by coronal T1 Turbo Inversion Recovery Magnitude (TIRM) (TR: 3350 ms, TE: 59 ms, TI: 230 ms) and axial T2 TIRM (TR: 4850 ms, TE: 30 ms, TI: 230 ms) to thoroughly evaluate subcutaneous tissue alterations, interstitial fluid distribution, and lymph node morphology.

Following image acquisition, post-processing was performed using the Siemens syngo Torus platform running Numaris/X (Version VA60A-OD4N, Software Release VB60).

Segment-Specific MIP: Maximum Intensity Projection (MIP) reconstructions were generated in the coronal plane for each anatomical station using a full-slab projection encompassing the acquired volume of the corresponding station. The anatomical coverage extended from the digits to the mid-calf for Station 1 (distal/pedal region), from the mid-calf to the mid-thigh, including the popliteal fossa, for Station 2 (crural/knee region), and from the mid-thigh to the pelvic girdle and inguinal region for Station 3 (femoral/pelvic region). These segment-specific reconstructions were used to visualize the distribution and anatomical continuity of fluid-sensitive structures within each station.

Panoramic Reconstruction: The datasets acquired at the three anatomical stations were combined using the vendor-native Syngo Inline Composing functionality (Siemens Healthineers). According to the described software workflow, automated rigid three-dimensional co-registration and intensity matching were applied using the geometric overlap between adjacent stations to generate a continuous panoramic reconstruction of the imaged lower-extremity regions.

**Figure 1:**
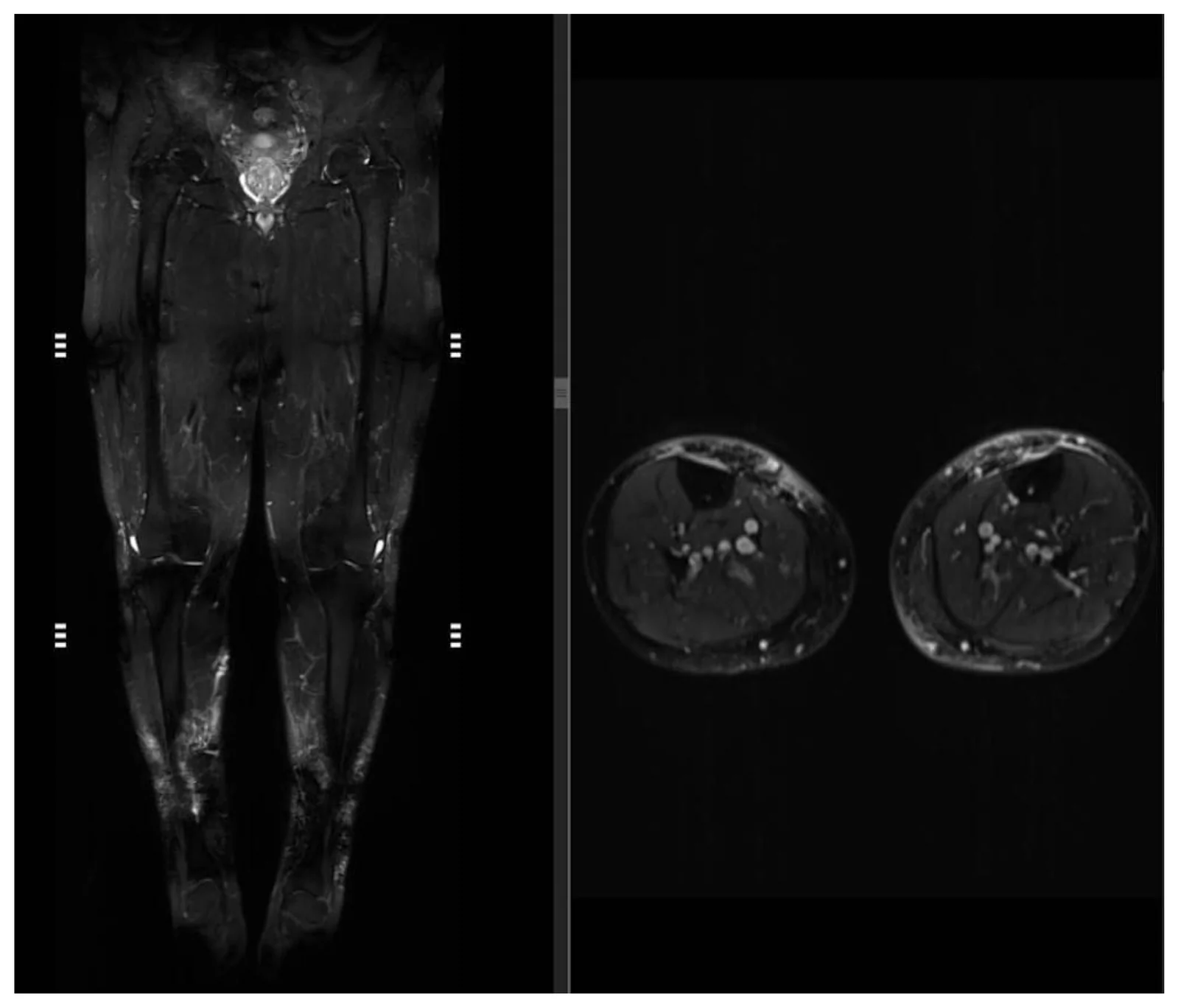
Coronal 3D T2 SPACE STIR panoramic view of the lower extremities obtained via multi-station image composition. Corresponding axial T2 TIRM image.

**Figure 2:**
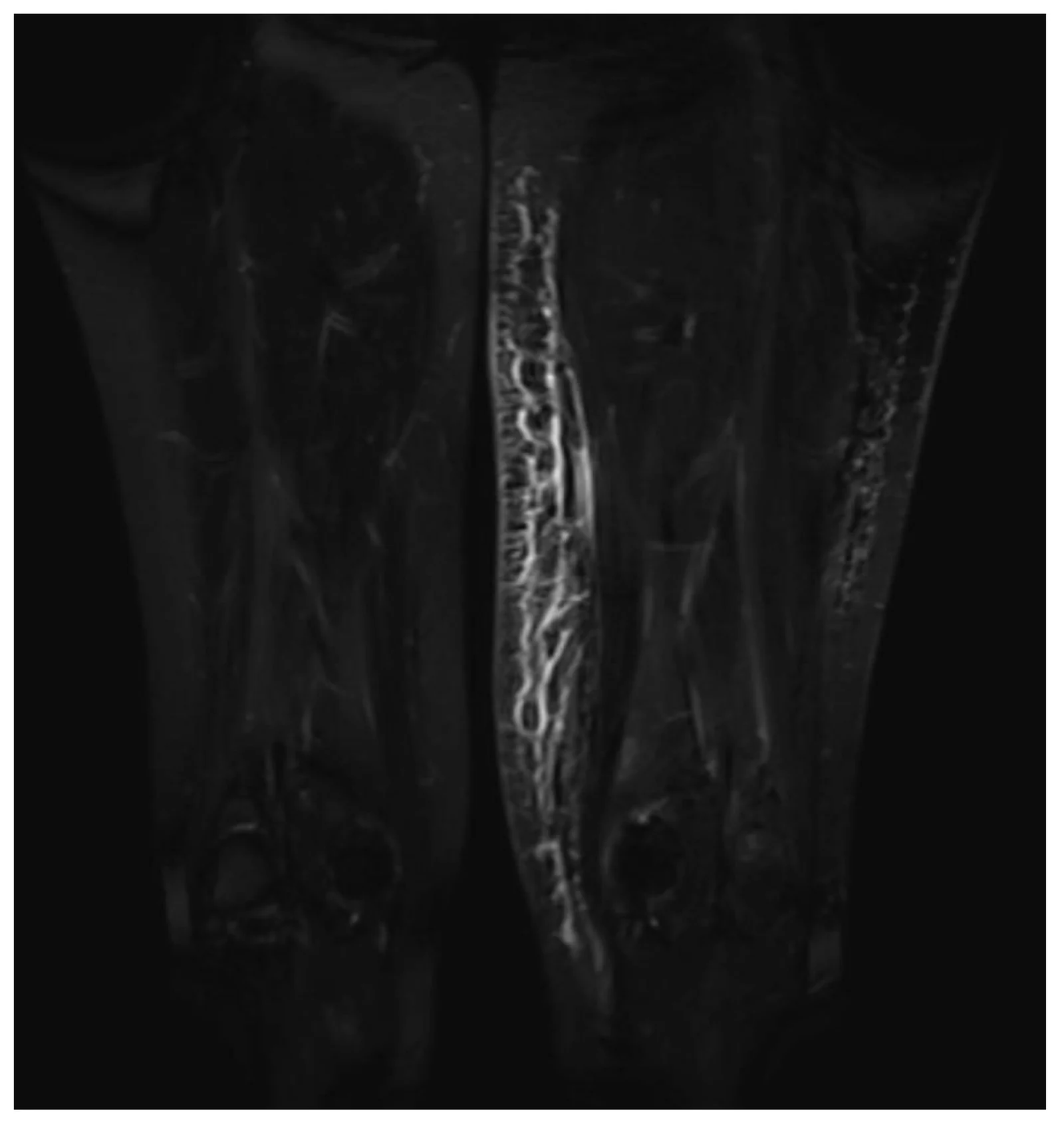
High-resolution coronal 3D T2 SPACE STIR targeted MIP reconstruction of the femoral region.

**Figure 3:**
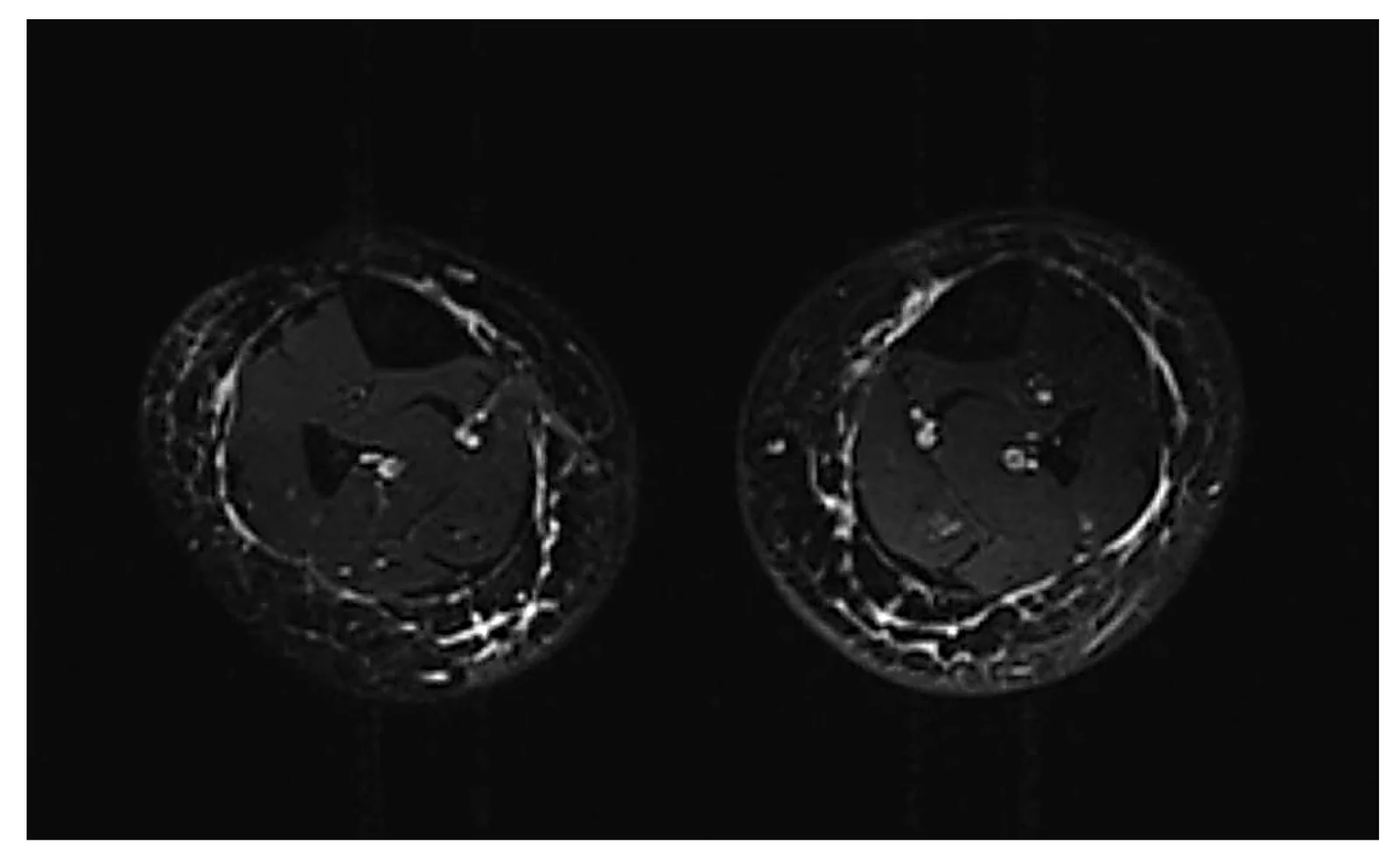
High-resolution cross-sectional axial T2 TIRM imaging at the mid-calf level.

**Figure 4:**
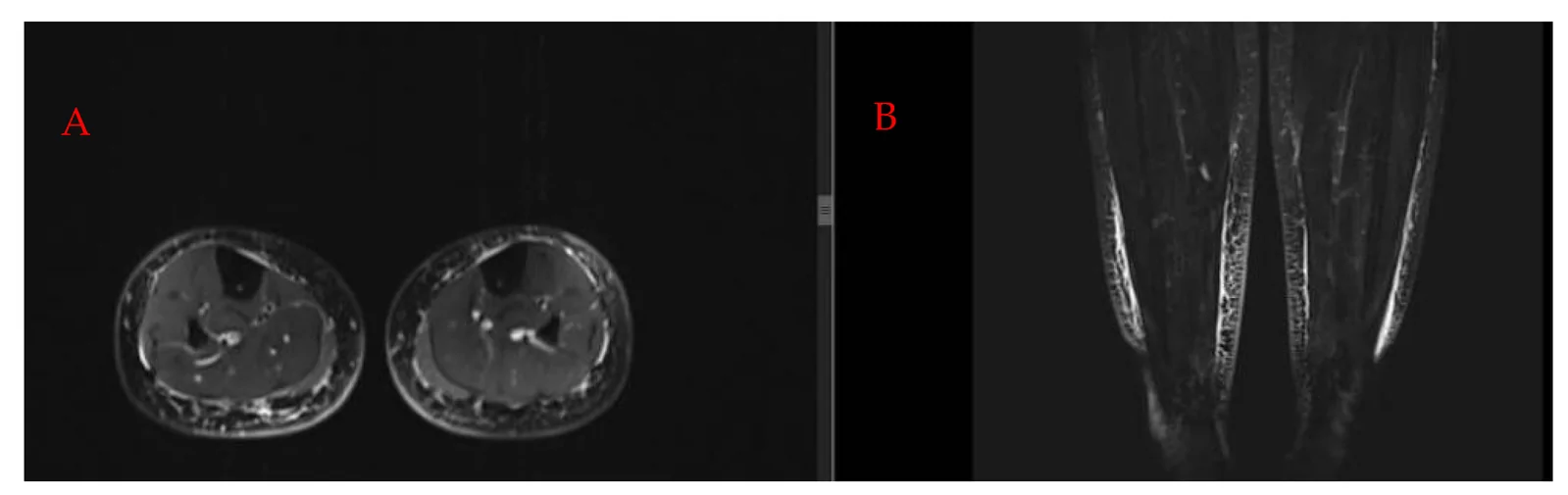
Multiplanar anatomical correlation of bilateral lower-extremity lymphedema using fluid-sensitive sequences. (**A**) Axial T2 TIRM cross-sectional view at the mid-calf level. (**B**) Corresponding coronal 3D T2 SPACE STIR image.

**Figure 5:**
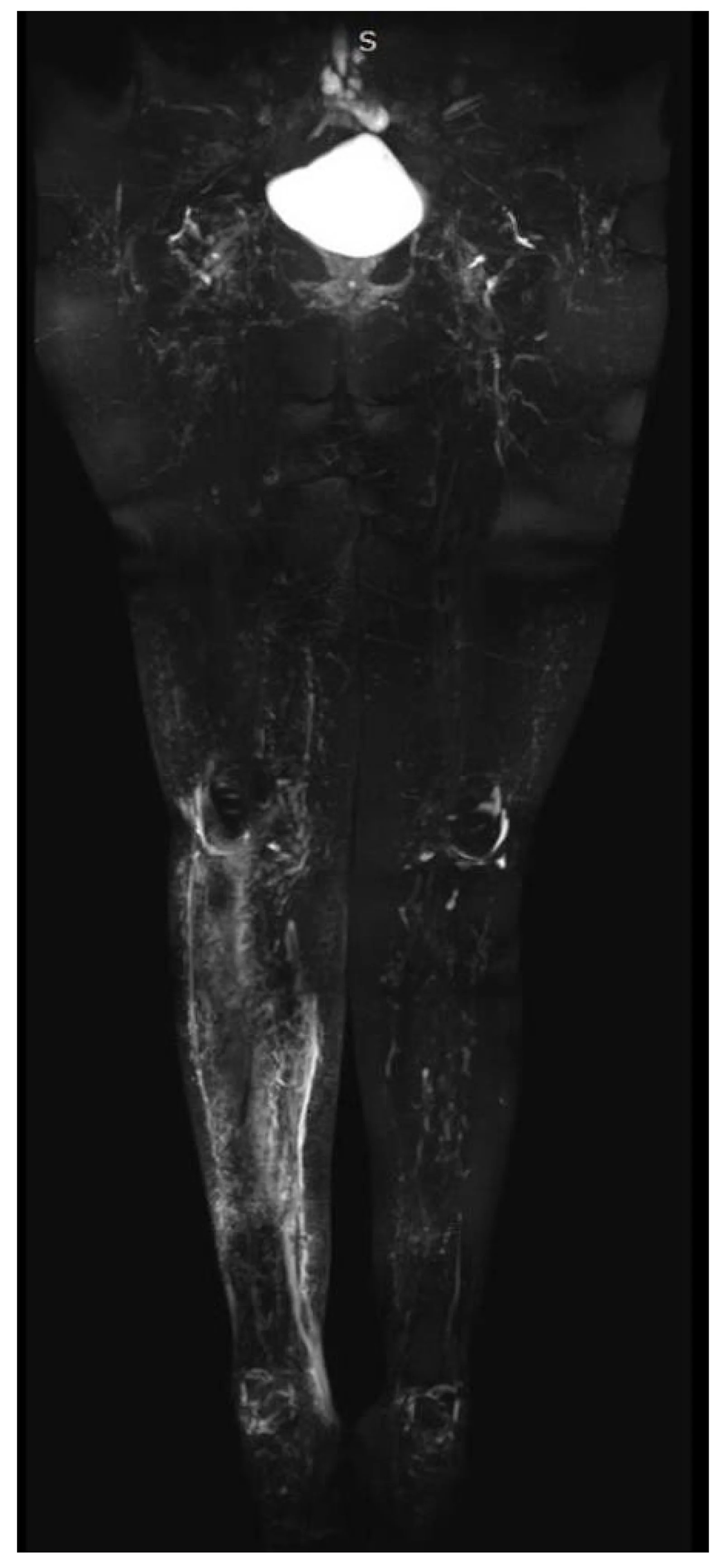
Representative panoramic 3D T2 SPACE STIR reconstruction obtained using the institutional NCMRL protocol, illustrating the anatomical coverage and image composition achieved through the described technical workflow.

Post-processing was performed without manual image editing or interactive manipulation of the acquired datasets. Only standard window and level adjustments were applied to optimize visual contrast (Table 1, Figure 1, Figure 2, Figure 3, Figure 4 and Figure 5).

The acquisition times reported in Table 1 refer to the individual MRI sequences and do not represent the total examination duration. Based on routine clinical experience rather than formal time measurements, the complete examination is estimated to take approximately 30–45 min, including patient positioning, MRI preparation, and image acquisition across the three anatomical stations.

## 3. Results

The technical output of the described institutional NCMRL workflow is illustrated by the representative images presented in the manuscript. These examples demonstrate the application of the three-station acquisition approach, the use of complementary imaging sequences, and the generation of segmental and panoramic MIP reconstructions.

As this manuscript is a descriptive technical report rather than a systematic clinical investigation, no predefined patient cohort was evaluated, and no formal assessment of examination success rates, diagnostic image adequacy, or patient-level clinical outcomes was performed. Consequently, quantitative feasibility estimates and diagnostic performance measures cannot be derived from the presented material.

## 4. Discussion

Advances in the diagnosis and treatment of cancer have led to a significant increase in patient survival and, consequently, in the number of long-term survivors [7]. As a result, the long-term complications of anticancer therapies are becoming increasingly clinically significant. Lymphedema is one of the most common complications, particularly following lymph node dissection [8]. Lymphedema is classified as primary and secondary, with cancer treatment recognized as the most common cause of secondary lymphedema in modern societies [9].

Due to the increasing incidence of lymphedema, it is essential to investigate it through appropriate diagnostic tests in order to confirm the diagnosis, assess the extent and severity of the disease, and design a personalized treatment plan tailored to each patient’s needs.

Various imaging methods have been used to evaluate the lymphatic system and diagnose lymphedema. Lymphoscintigraphy has long been considered the established gold standard for studying lymphatic function [2]. However, its limitations have led in recent years to increased research and clinical interest in magnetic lymphangiography, which offers more detailed imaging of the lymphatic system and new possibilities for the diagnosis and evaluation of lymphedema.

Although lymphoscintigraphy uses a radiolabeled tracer that is selectively taken up by lymphatic vessels (a fact that gives the method high specificity), it has limitations due to its low spatial and temporal resolution. Furthermore, it is usually limited to producing two-dimensional (2D) images of the major lymphatic pathways [3,4].

It has been reported that lymphoscintigraphy is associated with a small but real exposure of the patient to ionizing radiation [10]. Another disadvantage of lymphoscintigraphy is that subcutaneous lymphatic vessels cannot be adequately visualized, although the course and distribution of radiolabeled particles in lymphoscintigraphy are evaluated using a gamma camera [11].

On the other hand, there is intense research interest in magnetic lymphangiography. Contrast-enhanced magnetic resonance lymphangiography (CEMRL) is performed by subcutaneously injecting a gadolinium-based contrast agent into the interdigital spaces of the hands or feet [5,12]. It has been shown to be a safe technique that offers high spatial resolution and the ability to provide excellent visualization of lymphatic drainage [13].

However, CEMRL has certain restrictions. First of all, the use of a contrast agent may cause discomfort in patients and local reactions. Furthermore, there are findings suggesting that gadolinium is deposited in the brain following MRI scans [14]. In this context, efforts are being made to implement magnetic lymphography techniques that do not require the administration of a contrast agent, particularly in younger patients.

Non-contrast magnetic resonance lymphangiography offers the practical advantage of visualizing fluid-containing lymphatic structures without the administration of contrast agents. In our department, the total examination duration ranges from approximately 30 to 45 min. Unlike lymphoscintigraphy, which may require sequential imaging at different time points following radiopharmaceutical administration [15], NCMRL is performed without the use of radiopharmaceuticals or contrast agents. The absence of contrast administration avoids the associated risks and eliminates the need for contrast injection, potentially simplifying examination preparation [6]. These characteristics make NCMRL a useful imaging approach for the morphological assessment of the lymphatic system.

Nevertheless, non-contrast magnetic resonance lymphangiography has certain limitations. First, it is a relatively new imaging technique, and therefore its full potential and limitations in clinical practice have not yet been fully explored.

Furthermore, NCMRL is primarily based on the imaging of fluid-filled lymphatic structures and does not provide information regarding contrast uptake or the vascular characteristics of the tissues [6]. Thus, its exclusive use may not be sufficient in all cases for the complete evaluation of pathological findings, such as the differential diagnosis between benign and malignant lesions. Nevertheless, combined evaluation with other imaging and clinical methods can significantly contribute to a more accurate diagnosis and a more comprehensive assessment of the pathology.

Furthermore, the present technical report does not include a quantitative assessment of lymphatic drainage function, such as lymphatic flow velocity or drainage time, as the described NCMRL protocol is primarily intended for morphological imaging. In addition, the learning curve associated with protocol implementation and image interpretation, including operator training requirements and inter-operator variability, was not systematically evaluated. These aspects warrant further investigation in future studies.

Although NCMRL provides morphological information based predominantly on fluid-sensitive imaging, the present technical report does not establish its ability to reliably differentiate between specific pathological conditions, such as edema and fibrosis, lymphatic obstruction and hypoplasia, or primary and secondary lymphedema. Such distinctions require appropriate clinical correlation and, where indicated, complementary imaging techniques.

This study aims to contribute to the systematic documentation and description of the application of non-contrast magnetic lymphangiography, presenting how the technique is applied in our clinical practice. Through the analysis of these technical parameters and the methodology followed, we aim to contribute a foundational reference to the existing literature, supporting future efforts toward cross-vendor protocol standardization and optimization across different clinical centers. Although the described protocol was developed using a specific 3T MRI platform, its applicability to scanners from other manufacturers remains to be evaluated in future studies. Ultimately, establishing widely accepted protocols will help promote the broader clinical utility of non-contrast magnetic resonance lymphangiography.

### 4.1. Technical Context and the Need for Protocol Standardization

In recent years, there has been growing research interest in non-contrast magnetic resonance lymphography. What remains less well-established is the standardization of the imaging acquisition and processing procedures themselves, particularly in a way that allows for reproducibility and adaptability across different centers. This need is supported by the systematic review by Mills et al., which included 43 MRL studies, while the heterogeneity of MRL protocols is recognized as a significant limitation of the existing literature [5]. Although 58% of the studies used 3T systems and T2-weighted sequences were a common feature of the protocols, the authors concluded that further optimization and investigation of the impact of differences between protocols are needed [5].

Previous studies have investigated the diagnostic applications of NCMRL, including the characterization of morphological findings associated with lymphedema and their relationship with clinical staging [6,16,17]. In this context, the present report provides a structured description of our institutional 3T NCMRL workflow, incorporating predefined multi-station coverage, geometric alignment between sequences, complementary axial and coronal imaging, and segmental and panoramic MIP reconstructions. This approach is presented as a practical technical reference for future protocol harmonization and standardization.

### 4.2. Contribution of the Present Study

The contribution of the present study lies in the detailed documentation of an institutional technical workflow for NCMRL at 3T, which may serve as a reference for future protocol standardization efforts.

The proposed protocol incorporates predefined coverage of the lower extremities across three anatomical stations—pedal/distal crural, proximal crural/knee, and femoral-inguinal/pelvic—utilizing Body and Spine matrix coils simultaneously. This approach aims to provide extensive anatomical coverage through a structured acquisition workflow that may facilitate reproducibility in future investigations.

Particular emphasis is placed on maintaining consistent geometric settings and facilitating spatial correspondence between complementary imaging sequences. This approach supports anatomical correlation of findings between axial and coronal datasets. The fluid-sensitive findings demonstrated by this technique should be interpreted primarily as morphological imaging features rather than direct indicators of lymphatic flow or drainage function. This is significant, as contemporary reviews of lymphedema MRI highlight that axial sequences are particularly useful for assessing the extent and distribution of edema, whereas coronal 3D acquisitions offer more effective coverage of large anatomical areas [18].

The technical principles underlying NCMRL, including heavily T2-weighted fluid-sensitive imaging and MIP reconstruction, have been described in previous publications, including those by Mills, Arrivé, and Franconeri [5,16,17]. The contribution of the present report is not the introduction of a novel imaging sequence or reconstruction method, but the detailed documentation of how established techniques are integrated into a structured institutional 3T workflow. This includes three-station anatomical coverage, complementary axial and coronal acquisitions, and segment-specific and panoramic post-processing.

### 4.3. Technical Value of Multiplanar Imaging, Geometric Co-Registration, and Post-Processing

The proposed protocol combines panoramic heavily T2-weighted reconstructions with three-dimensional coronal and axial imaging and sequential post-processing.

As shown in Figure 1, the composed coronal 3D T2 SPACE STIR reconstruction provides a comprehensive panoramic view of the lower extremities, while the corresponding axial T2 TIRM allows for a more detailed assessment of the transverse distribution of changes in the subcutaneous tissues. Panoramic imaging is particularly useful for assessing the cranial-caudal extent of lesions, symmetry between the two limbs, and the overall distribution of high-signal structures. However, a single projection or panoramic image cannot always accurately depict the relationship of a finding to the skin, subcutaneous adipose tissue, deep fascia, and muscle compartments. For this reason, correlating 3D coronal data with transverse images is an essential part of the interpretation and not just additional imaging information.

Figure 3 and Figure 4 demonstrate the complementary information provided by axial and coronal imaging. High-resolution axial T2 TIRM imaging at the level of the lower legs allows for a direct comparison of the two limbs and an assessment of fluid distribution within the subcutaneous compartment, while correlating the same anatomical region with coronal 3D T2 SPACE STIR imaging enables the evaluation of the same finding in a different plane.

Additionally, the proposed protocol incorporates two distinct levels of MIP post-processing, each serving different but complementary purposes. First, a segment-specific MIP reconstruction is performed for each anatomical station, and then the individual datasets are combined to create global composed panoramic reconstructions. Figure 2, which shows a segment-specific MIP reconstruction of the femoral region, is a typical example of the first approach. The limited anatomical coverage of the segment-specific MIP allows for a more focused assessment of high-signal structures and reduces the anatomical overlap that can occur when a large portion of the limb is displayed in a single image. In contrast, global composed reconstruction provides an overall view of the distribution of findings along the lower extremities. The two forms of reconstruction should therefore not be considered competitive.

Finally, Figure 5 provides a representative example of the panoramic reconstruction obtained using the proposed institutional workflow, illustrating the technical implementation of the acquisition and post-processing procedure. A more broadly applicable standardization framework could include, in addition to basic acquisition parameters, predefined quality criteria for the final image.

From this perspective, Figure 1, Figure 2, Figure 3, Figure 4 and Figure 5 serve not only as representative examples of imaging findings but also illustrate successive stages of the proposed NCMRL workflow: ranging from multi-station acquisition and complementary axial/coronal imaging to the geometric registration of distinct datasets, local MIP assessment, and, ultimately, the generation of a comprehensive panoramic reconstruction. This workflow-based approach can serve as a practical foundation for the future definition of both minimum technical requirements and quality criteria for NCMRL.

The contribution of this study does not concern the introduction of NCMRL as a new imaging technique or the use of sequences with strong T2 weighting or MIP reconstructions, which have already been described. Instead, its main value consists of documenting the integration of specific technical elements into a structured institutional 3T workflow, with particular attention to patient positioning, anatomical coverage, sequence geometry, and post-acquisition processing. Future validation of these principles across various MRI systems, manufacturers, and patient populations could represent the next step toward the development of widely accepted NCMRL protocols.

### 4.4. Limitations

The present report is descriptive and technical in nature. Quantitative image quality assessment, inter-observer and intra-observer reliability, and cross-vendor reproducibility were not evaluated. These aspects require further investigation before the proposed workflow can be considered a validated standardized protocol.

Furthermore, the described protocol was not designed to provide quantitative assessment of lymphatic flow dynamics or drainage function, and its findings should not be considered equivalent to those obtained from dedicated functional lymphatic imaging techniques.

Accordingly, the representative images presented in this report should be interpreted solely as illustrations of the described acquisition and reconstruction workflow, rather than as evidence of diagnostic accuracy, clinical effectiveness, or validated reproducibility.

## 5. Conclusions

Non-contrast magnetic resonance lymphangiography represents a promising imaging technique for the morphological evaluation of the lymphatic system, enabling anatomical visualization without the administration of contrast agents.

However, heterogeneity among existing imaging protocols remains a challenge for reproducibility and broader clinical implementation. This paper provides a detailed description of the technique and acquisition parameters applied in routine clinical practice at our institution, contributing to the documentation and dissemination of existing technical experience. Through this descriptive technical contribution, we aim to support ongoing efforts toward the optimization, harmonization, and future standardization of lymphatic imaging protocols.

Further systematic investigations are required to evaluate the diagnostic performance, reproducibility, and broader clinical applicability of the described workflow.

## Figures and Tables

**Table 1:** MRI Sequence Parameters for non-contrast magnetic resonance lymphangiography (NCMRL).

Sequence Type	Plane	TR/TE (ms)	Slices/Slabs	Slice Thickness (mm)	TI (ms)	FA	BW (Hz/Px)	FOV * (mm)	Acquisition Matrix	Scan Time
3D T2 SPACE STIR	Coronal	4000/350	64/1 slab	3.0	200	T2 VAR	698	450	301 × 448	4:20 Min
T2 TIRM	Axial	4850/30	50 slices	4.0	230	124°	246	340–360	378 × 384	3:16 Min
T1 TIRM	Coronal	3350/59	40 slices	4.0	230	134°	252	400–450	230 × 320	3:43 Min

* Note: Field of View (FOV) parameters were dynamically adjusted and tailored across all sequences based on individual patient habitus, stature, and structural somatotype to maximize anatomical coverage and signal homogeneity. T2 VAR: Variable Flip Angle application optimized for T2 contrast. BW: bandwidth; FA: flip angle. Scan times are expressed in minutes and seconds (min) and refer to individual sequence acquisitions, not to the total examination duration.

## Data Availability

The original contributions presented in this study are included in the article. Further inquiries can be directed to the corresponding author.

## Abbreviations

NCMRL	Non-Contrast Magnetic Resonance Lymphography
CEMRL	Contrast-Enhanced Magnetic Resonance Lymphography
MRL	Magnetic Resonance Lymphography
MRI	Magnetic Resonance Imaging
MIP	Maximum Intensity Projection
STIR	Short Tau Inversion Recovery
TIRM	Turbo Inversion Recovery Magnitude
SPACE	Sampling Perfection with Application-optimized Contrasts using different flip angle Evolutions
FOV	Field of View
TR	Repetition Time
TE	Echo Time
TI	Inversion Time
FA	Flip Angle
BW	Bandwidth
SNR	Signal-to-Noise Ratio
CNR	Contrast-to-Noise Ratio
B0	Main Magnetic Field
B1	Radiofrequency Magnetic Field
FFS	Feet First Supine
